# Topical imiquimod in the management of botulinum toxin-induced upper eyelid ptosis: A case series

**DOI:** 10.1016/j.jdcr.2026.02.024

**Published:** 2026-02-17

**Authors:** Simona Abou Tayeh, Ribal Merhi, Maria Joy Khachan, Cristelle Obeid, Nakhle Ayoub

**Affiliations:** a School of Medicine and Medical Sciences, Holy Spirit University of Kaslik, Jounieh, Lebanon; b Service de dermatologie (Department of Dermatology), Hôpital Saint André, CHU de Bordeaux, Bordeaux, France; c Dermatology Department, Hôtel-Dieu de France, Beirut, Lebanon

**Keywords:** abobotulinum toxin A, botulinum toxin type A, imiquimod, incobotulinum toxin A, onabotulinum toxin A, TLR7 agonist, upper eyelid ptosis

## Introduction

Botulinum toxin is an injectable neuromodulator derived from the anaerobic, spore-forming, gram-positive bacillus bacterium *Clostridium botulinum*, which inhibits acetylcholine release from presynaptic cholinergic neurons. To date, 8 distinct serotypes (A-H) have been identified, with serotypes A and B approved for clinical use. The US FDA first approved it in 1989 for blepharospasm, hemifacial spasm, and strabismus, followed by approval for cervical dystonia in 2000 and cosmetic treatment of glabellar wrinkles in 2002.^1^ Botulinum neurotoxin type A (BoNT-A) has been proven safe since then, with rare adverse events such as bleeding, bruising, swelling, erythema. Other side effects include ectropion near the lower eyelid, strabismus when treating the lateral canthal rhytids or transverse nasal rhytids, and most commonly, eyelid ptosis (blepharoptosis) following glabella and/or frontalis injections.

Blepharoptosis is a common adverse effect of botulinum toxin type A (BoNT-A), defined as drooping of the upper eyelid due to diffusion of the toxin to the levator palpebrae superioris (LPS) within the superior intraorbital compartment.^2^ The superficial frontal (third facial layer) and deep intraorbital (fifth facial layer) regions are separated by the orbital septum, whose fibrous insertions at the superior orbital rim can permit toxin spread via the supratrochlear, supraorbital, or lacrimal neurovascular pedicles.^2^ This results in partial or complete eyelid drooping, a poor cosmetic result that may interfere with normal vision.

Ptosis is diagnosed when the upper eyelid lies 1.5 to 2 mm below the limbus.^3^ BoNT-A-induced ptosis is myogenic, resulting from transient neuromuscular transmission blockade. Symptoms usually appear 2 to 10 days post-injection and may persist for the full duration of toxin activity. Steinsapir et al reported 7 cases with persistent ptosis lasting about 6 to 13 weeks.^4^

Although management is typically conservative awaiting spontaneous resolution over weeks to months, various treatments have been described in the literature, including topical α-adrenergic agonists, oral anticholinesterase and targeted botulinum toxin injections into the pre-tarsal orbicularis.

Topical imiquimod 5% cream is a toll-like receptor 7 (TLR7) agonist. Imiquimod has long been established in dermatology as a potent immune modulator for conditions such as actinic keratoses, basal cell carcinoma, and lentigo maligna. Imiquimod binds TLR-7 on dendritic cells, macrophages, and monocytes, triggering release of IFN-α, TNF-α, IL-1, IL-6, IL-8, IL-10, and IL-12. These cytokines drive the activation of the adaptive immune response toward the TH-1 or cell-mediated pathway and inhibit the TH-2 pathway, induce antiviral mechanisms including 2′,5′-oligoadenylate synthetase and NK-cell activation, and enhance Langerhans-cell maturation/migration to regional lymph nodes, improving antigen presentation to naïve T cells.^5^

We hypothesized that topical immune stimulation may accelerate toxin neutralization or clearance at the neuromuscular junction. This hypothesis emerged after several of our patients reported unusual fast fading of botulinum toxin effect after using Imiquimod for the treatment of facial plane warts. We report a case series of 4 patients who developed post-injection upper-eyelid ptosis and experienced unusually rapid recovery after localized application of topical imiquimod 5% cream to the affected frontalis/glabellar region.

## Case 1

A 49-year-old woman with a history of well-controlled hypertension presented with new-onset left upper-eyelid ptosis that developed 5 days after a cosmetic botulinum toxin procedure to the glabellar complex and upper forehead performed at a private aesthetic center. The product and dose used were not documented.

**Fig 1:**
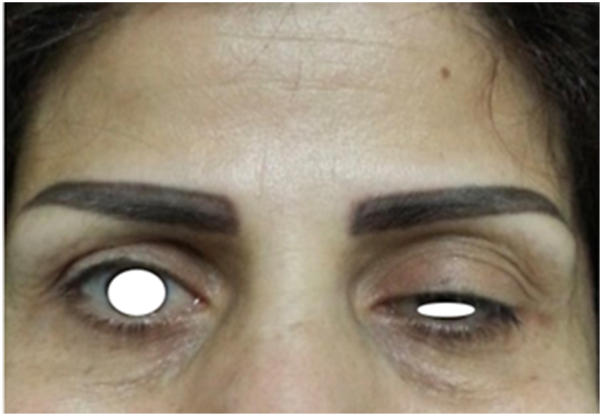
Baseline appearance showing left upper-eyelid ptosis 5 days after botulinum toxin injection.

On examination, the left upper eyelid margin rested approximately 5 mm lower than the right, partially covering the superior limbus and decreased left frontalis activity with preserved contralateral movement (Fig 1).

However, because ocular α-adrenergic agonists such as apraclonidine were contraindicated in her case due to hypertension, alternative management was considered. The patient was instructed to apply topical imiquimod 5% cream (Aldara) once daily over the left upper forehead, while avoiding the eyelid and periorbital skin.

**Fig 2:**
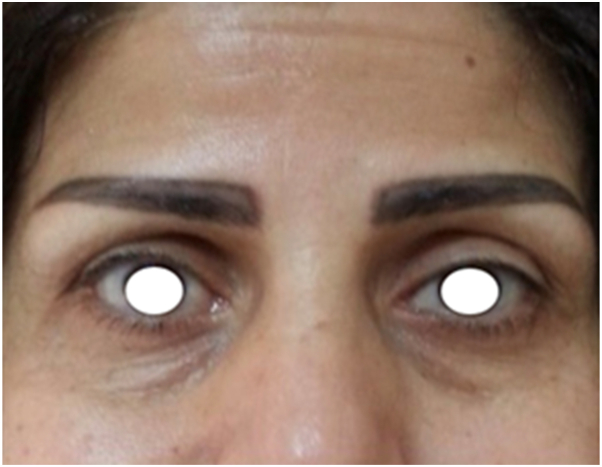
Follow-up 48 hours after topical imiquimod 5% application to the left forehead.

Remarkably, within 48 hours of initiation, the patient noticed spontaneous reopening of the affected eyelid and restoration of brow elevation (Fig 2). At the follow-up visit on day 3, examination confirmed complete resolution of ptosis and return of forehead rhytids over the treated area, indicating restoration of frontalis contractility.

Treatment was discontinued after a week with sustained improvement on subsequent follow-up. No adverse cutaneous reactions were observed.

## Case 2

A 49-year-old woman presented for evaluation of unexpected upper eyelid ptosis following botulinum toxin injection. She had undergone multiple cosmetic sessions with abobotulinumtoxin A (Dysport) at our clinic over the preceding 3 years with consistently satisfactory results. Her medical history includes no prior neurologic or dermatologic conditions.

This patient presented with left upper-eyelid ptosis that developed 4 days after receiving botulinum toxin injections at an unlicensed beauty salon. She reported a feeling of heaviness of the left brow and mild asymmetry when raising her eyebrows. Examination revealed reduced left frontalis activity.

Imiquimod 5% cream was applied daily to the left upper forehead and improvement was noted within 72 hours, with complete recovery of brow elevation and reduction of ptosis. No adverse cutaneous reactions occurred.

Videos 1 and 2 are available as Supplementary Material, available via Mendeley at https://data.mendeley.com/datasets/ph83gnpt5k/1.

## Case 3

A 69-year-old woman presented with mild left-sided eyelid drooping that developed 1 week after receiving onabotulinum toxin A (Allergan), injections to the glabellar complex (procerus and corrugator muscles) and upper forehead (frontalis). The total injected dose was 38 units, distributed symmetrically. Her medical history included well-controlled hypertension. On examination, the left upper eyelid margin rested 2 mm lower than the right. This patient was prescribed topical imiquimod 5% cream applied twice weekly for 3 weeks to the left upper forehead region, avoiding the orbital rim.

By day 7 of imiquimod application twice weekly, examination revealed full restoration of right frontalis function. No adverse cutaneous reactions occurred.

Videos 3 and 4 are available as Supplementary Material, available via Mendeley at https://data.mendeley.com/datasets/ph83gnpt5k/1.

## Case 4

A 55-year-old woman with 2 prior uneventful sessions of abobotulinumtoxin A (Dysport) at our clinic presented with acute left upper-eyelid ptosis that appeared 4 days after receiving an unknown botulinum toxin formulation from an unlicensed practitioner. The injection reportedly involved the glabellar region and forehead, though precise dosing and dilution were not documented. The patient described a sensation of heaviness over the left brow. Her past medical history included no neurologic or autoimmune disorders.

On examination, the left upper eyelid margin covered the superior limbus by approximately 3 mm. Topical imiquimod 5% cream was prescribed once daily, applied to the left upper forehead and glabellar region, sparing the eyelid margin.

After 5 days, objective improvement was evident, with restoration of symmetric frontalis contraction and complete resolution of ptosis by day seven. No irritation, erythema, or systemic symptoms were reported.

Videos 5 and 6 are available as Supplementary Material, available via Mendeley at https://data.mendeley.com/datasets/ph83gnpt5k/1.

## Discussion

Upper eyelid elevation is mediated by 2 muscles: the levator palpebrae superioris muscle (LPS) and superior tarsal muscle (STM), or Müller's muscle.^6^ The LPS—antagonistic to orbicularis oculi and innervated by the superior branch of CN III—inserts into the superior tarsal plate/eyelid skin at the orbital septum and raises the lid ∼12 to 20 mm; STM adds ∼2.5 mm via sympathetic tone.^6^ The underlying mechanism for BoNT-A-induced ptosis is based on the effect of the toxin on nerve terminals of the muscles which the practitioner does not intend to target. The patient with eyelid ptosis has toxin bound to nerve endings of an unintended targeted muscle as well. The unintended targeted muscle in blepharoptosis is the LPS, as the STM adrenergic muscle is not as accessible by BoNT-A.

Risk is governed by injection technique (site, muscle mass, reconstitution/injection volume, depth, speed of injection, needle gauge); medial injections into lower frontalis/orbicularis oculi (medial to the mid-pupillary line) may spread through the orbital septum via the pre-periosteal plane or superior ophthalmic vein tributaries to the LPS.^7^ The lower the spread of toxin, the higher the accuracy in treating intended targets and the lower the chance of eliciting side effects such as ptosis. Risk factors for eyelid ptosis also include patient characteristics such as older age, sun-damaged skin, decreased elasticity, a heavy or short brow, and outdoor work, which increase frontalis dependence and vulnerability to drooping when muscle activity is reduced.^8^ Patients with a history of facial surgery, preexisting ptosis, Bell’s palsy, or neuromuscular disorders like myasthenia gravis or multiple sclerosis should be approached cautiously due to altered muscle function or neuromuscular transmission issues. Factors related to the product, such as improper dilution or poor quality, may cause unpredictable toxin spread and inconsistent outcomes. Adopting a conservative injection strategy—superficial placement with low volumes and avoidance of deep injections that could involve the supraorbital nerve or superior ophthalmic venous branches reduces diffusion and neurovascular risk.

Management of eyelid ptosis is primarily supportive and symptomatic. Oxymetazoline ophthalmic solution 0.1% (UPNEEQ), a topical α-adrenergic agonist, received FDA approval in 2020 for acquired blepharoptosis and provides a topical, targeted option.^9^ Before oxymetazoline was available, apraclonidine 0.5% drops were commonly used for botulinum toxin–induced ptosis. However, apraclonidine can cause mydriasis and is more often associated with ocular irritation or contact dermatitis.^10^ Systemic and local alternatives for BoNT-A–related ptosis include oral anticholinesterases, such as pyridostigmine.^11^ Additionally, targeted botulinum toxin injections into the pre-tarsal orbicularis have been used to correct small asymmetries.^12^

Imiquimod is a potent inducer of localized, robust inflammatory responses via Toll-like receptor 7 (TLR-7) activation. It has been repeatedly shown to increase expression of proinflammatory cytokines (TNF-α, IL-1β, IL-6), chemokines, IFN-α, and immune cell infiltration in skin (keratinocytes, Langerhans/dendritic cells, plasmacytoid DCs) in both animal and human studies. Imiquimod-triggered inflammation might accelerate the neutralization, breakdown, or clearance of BoNT-A that has diffusely invaded non-target tissues, or it might enhance the local tissue’s repair pathways by recruiting immune cells and increasing vascular/lymphatic activity. For example, increased local cytokine production could enhance recruitment and activation of phagocytic cells or up-regulate local proteases that degrade extracellular or synaptic protein complexes, thereby reducing the effective concentration of active toxin. One compelling rationale for considering imiquimod in BoNT-A–induced eyelid ptosis is that inflammation, while often seen as detrimental (for example UV-induced inflammation undermines BoNT-A effects), may in fact be leveraged therapeutically to counteract unwanted toxin spread. Although narrowband UVB phototherapy is widely used for its cumulative anti-inflammatory effects in dermatologic conditions, acute UVB exposure initially triggers a classic pro-inflammatory response characterized by erythema, cytokine release, and leukocyte infiltration. In contrast, chronic or repeated UVB exposure can induce immunomodulatory and suppressive changes in cutaneous immune pathways. Indeed, genomic studies have shown distinct molecular differences between acute and chronic UVB exposures, with acute UVB activating inflammatory signaling and chronic exposure promoting immune downregulation and tolerance.^13^ This concept is further supported by Sycha et al who conducted a randomized, double-blind, placebo-controlled human study that demonstrated that UV-B irradiation following intradermal BoNT-A reduced the BoNT-A anhidrotic (sweat-blocking) area by approximately 30% over 14 weeks, demonstrating that cutaneous inflammation and UV exposure can significantly attenuate BoNT-A’s functional blockade.^14^ Such time-dependent effects of UVB on inflammation and immunity help resolve the apparent paradox between its immediate pro-inflammatory actions and its longer-term anti-inflammatory or immunosuppressive therapeutic effects. Because BoNT-A effect depends on persistence of toxin at its site of action, accelerated degradation (via proteolytic, oxidative, or immune-mediated mechanisms) might shorten duration of ptosis. Importantly, when used peri-ocularly (for eyelid or peri-ocular basal cell carcinoma, lentigo maligna, etc.), it has been shown to achieve good efficacy with generally mild to moderate, transient local adverse effects such as erythema, crusting, edema; serious ocular complications are rare and reversible.^15^ The recovery timeline following imiquimod application—significantly shorter than the expected 4 to 8 weeks for spontaneous resolution—suggested a true pharmacologic reversal of toxin effect.

Alternative explanations must be considered. Spontaneous recovery remains possible, and placebo or observer effects cannot be excluded in an uncontrolled case series. Notably, the magnitude and rapidity of recovery in our patients, the anatomic specificity of the response (reappearance of forehead rhytids confined to the imiquimod application area), and the recurrence of the phenomenon in a single patient after 2 separate exposures argue against pure chance. Nevertheless, a causal relationship cannot be established from case series alone. The type, dose, and technique of the antecedent botulinum-toxin injections were heterogeneous and, in some cases, undocumented, and the imiquimod application regimens were not standardized. Objective eyelid measurements and blinded photographic assessment were not consistently available, and the possibility of spontaneous recovery or placebo effect cannot be excluded. These limitations underscore the need for prospective, controlled studies with standardized interventions, objective outcome measures, predefined safety monitoring, and mechanistic investigations.

## Conclusion

Botulinum toxin A is a powerful neuromodulator whose cosmetic effects depend on temporary paralysis of facial muscles. Although eyelid ptosis is relatively rare when injections are done by clinicians, the risk is substantially higher in less experienced hands. Imiquimod, a potent TLR-7 agonist, induces localized immune activation, upregulating cytokines (TNF-α, IL-1β, IL-6) and recruiting immune cells, potentially facilitating clearance of diffused BoNT-A and neuromuscular junction repair. Given evidence that inflammation can reduce BoNT-A activity and the tolerability of imiquimod near the eyes, imiquimod is a biologically plausible adjunct for BoNT-A-induced upper eyelid ptosis. Prospective trials are needed to define optimal dosing, timing, safety, and clinical efficacy.

## Conflicts of interest

None disclosed.
